# Supplementary material for: Phytoconstituents and Ergosterol Biosynthesis-Targeting Antimicrobial Activity of Nutmeg (Myristica fragans Houtt.) against Phytopathogens
Source: Molecules. 2024 Jan 18;29(2):471. doi: 10.3390/molecules29020471 (PMC10819938; doi:10.3390/molecules29020471)
Supplement: Supplementary file 1 [file molecules-29-00471-s001.zip › molecules-2825206-supplementary.pdf]

Phytoconstituents and Ergosterol Biosynthesis-Targeting Antimicrobial Activity of Nutmeg (*Myristica fragans* Houtt.) against Phytopathogens

Adriana Cruz [1,2], Eva Sánchez-Hernández [3], Ana Teixeira [1,4], Rui Oliveira [1,2], Ana Cunha [1,2], and Pablo Martín-Ramos [3,*]

[1] Department of Biology, School of Sciences, University of Minho, Campus de Gualtar, 4710-057 Braga, Portugal; cruzadriana73@gmail.com (A.C.).
[2] Centre of Molecular and Environmental Biology (CBMA), University of Minho, Campus de Gualtar, 4710-057 Braga, Portugal; accunha@bio.uminho.pt (A.C.); ruipso@bio.uminho.pt (R.O.).
[3] Department of Agricultural and Forestry Engineering, ETSIIAA, Universidad de Valladolid, Avenida de Madrid 44, 34004 Palencia, Spain; eva.sanchez.hernandez@uva.es (E.S.-H.), pmr@uva.es (P.M.-R.).
[4] Centre for Research and Technology of Agro-Environmental and Biological Sciences (CITAB), Inov4Agro, University of Trás-os-Montes and Alto Douro (UTAD), Quinta de Prados, 5000-801 Vila Real, Portugal; anaspereirateixeira@gmail.com (A.T.).
[*] Correspondence: pmr@uva.es

SUPPLEMENTARY MATERIAL

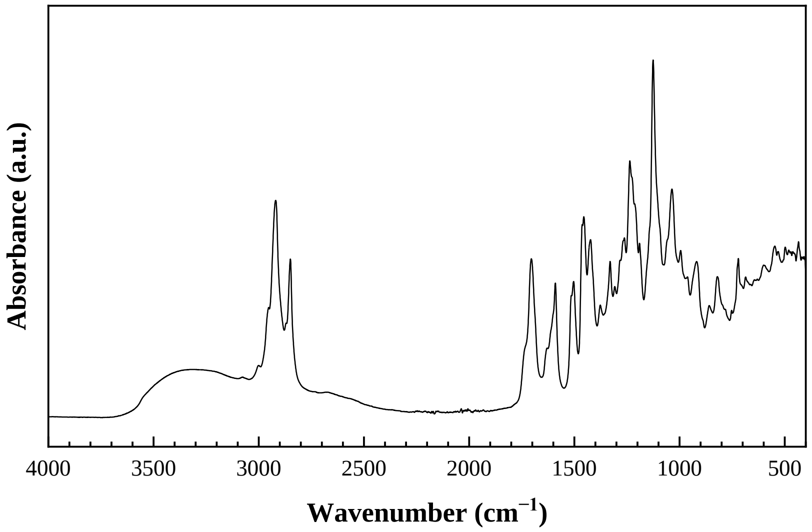

Figure S1. ATR–FTIR spectrum of the freeze-dried *M. fragrans* hydroethanolic extract

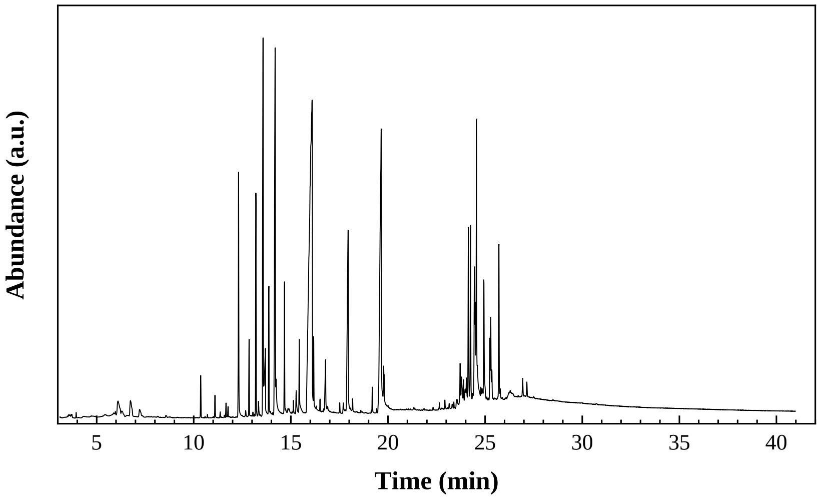

Figure S2. GC–MS chromatogram of *M. fragrans* hydroethanolic extract.
